# Low levels of serum vitamin C in children with limb fractures: a case-control study

**DOI:** 10.3389/fped.2023.1226508

**Published:** 2023-09-12

**Authors:** Yangkun Ding, Chunhua Wang, Jiazhi Yu, Mingzhu Lu, Pengfei Mu, Xiangfei Liu, Tao Liu

**Affiliations:** Department of Pediatric Orthopedics, Jinan Children’s Hospital, Jinan, China

**Keywords:** vitamin C, ascorbic acid, serum, fracture, pediatric

## Abstract

**Introduction:**

The role of vitamin C in pediatric fractures has not received much attention, although it is known to be a factor in osteoporotic fractures in the elderly. This case-control study aimed to investigate the changes in serum vitamin C levels among children with limb fractures.

**Methods:**

We recruited 325 children with and 316 children without limb fractures hospitalized between January 2021 and December 2021. Following admission, basic demographic data of all participants were collected, and fasting serum vitamin C levels were determined using ultra-high-performance liquid chromatography-tandem mass spectrometry.

**Results:**

The mean age of the fracture group was 5.1 years (95% CI, 4.83–5.33). The serum vitamin C levels in the fracture group (4.48 µg/ml) were significantly lower than those in the control group (8.38 µg/ml) (*p* < 0.0001). Further subgroup analysis of the fracture group revealed that serum vitamin C levels decreased significantly after 4 years of age and there was a significant difference in the duration after injury between <6 and >6 h (*p* = 0.0224). Spearman’s rank correlation coefficient suggested that age and vitamin C levels were negatively correlated in the fracture group.

**Conclusion:**

In general, children with limb fractures had lower serum vitamin C levels, especially those aged 4 years and over.

## 1. Introduction

Vitamin C, also known as ascorbic acid, is indispensable for bone collagen synthesis and an important antioxidant that can alleviate oxidative stress responses (1, 2). Owing to the absence of the L-gluconolactone oxidase enzyme in the liver, the human body cannot synthesize vitamin C (2, 3). Thus, if supplementation is not provided in the daily diet, it will eventually lead to a vitamin C deficiency (4).

The relationship between vitamin C and fractures has attracted widespread attention (5, 6). The role of vitamin C in bone metabolism is notable as it is associated with the hydroxylation of collagen and the expression of non-collagenous proteins, such as alkaline phosphatase, osteonectin, and osteocalcin. Vitamin C also promotes the expression of genes related to osteoblastogenesis and osteoclastogenesis via the Wnt/β-catenin/ATF4 signaling pathways (7–9). Preclinical and clinical studies have shown that vitamin C deficiency inhibits collagen synthesis and decreases bone formation. In vivo and *in vitro* studies have shown that vitamin C deficiency stimulates osteoclastogenesis by upregulating the RANKL/RANK pathway, and inhibits osteoblastogenesis by decreasing pro-collagen I mRNA expression and hydroxylation of collagen fibers (7–11) Thus, this deficiency has been a critical risk factor in osteoporotic fractures (11, 12). As the elderly require increased amounts of vitamin C, most studies have focused on the role of vitamin C status in fractures in elderly populations (10, 11, 13). It has been shown that serum vitamin C levels are significantly lower in older adults with fractures caused by low-impact injuries (13, 14).

Limb fractures are common in the pediatric trauma setting, including supracondylar humerus, forearm, femoral shaft, and tibial fractures (15, 16). Many studies have addressed that children with limb fractures are frequently deficient in vitamin D (17, 18); several reports exist on spontaneous fractures in infancy and young children with scurvy (19). Nevertheless, limited clinical data exist targeting the variation of vitamin C in children with limb fractures caused by trauma.

In this study, we investigated serum vitamin C levels in children with limb fractures and compared them with those of other patients. Additionally, we explored the factors that influence serum vitamin C levels among patients with fractures.

## 2. Materials and methods

### 2.1. Participants

In this case-control study, 715 participants were hospitalized at the authors’ hospital between January 2021 and December 2021. In the fracture group, qualified patients met the following inclusion criteria: (1) age ≤14 years; (2) first fracture; (3) duration after injury ≤24 h; (4) mild or moderate trauma according to Clark’s classification (16); and (5) all fractures surgically treated. Conditions excluded from the study included fractures related to bone metabolic diseases, long-term drug use affecting bone metabolism, pathological fractures, congenital diseases, fatigue fractures, and neuromuscular diseases.

The control group consisted of 364 hospitalized patients without fractures during the same period. In addition, these patients had no history of fractures for at least 1 year. Those with bone metabolic diseases, neuromuscular diseases, tumors, infectious diseases, congenital skeletal deformities, and long-term use of drugs that affect bone metabolism were excluded.

The study was conducted in accordance with the Declaration of Helsinki and approved by the Ethics Committee of the Institutional Review Board of the authors’ hospital. Informed consent was obtained from all participants included in the study and one parent or guardian of each child.

### 2.2. Data collection and evaluation

Basic demographic data of all participants, including sex, age, height, weight, body mass index (BMI), and clinical diagnosis were collected. Additionally, information on fractures, such as the type of injury, duration of time after injury, and fracture site, was recorded.

Venous blood was collected from each participant immediately after admission, and the serum concentrations of vitamin C were analyzed using ultra-high-performance liquid chromatography-tandem mass spectrometry. The simple steps were as follows: 1 ml of fasting venous blood was collected, and the blood was slowly poured into a coagulation tube, centrifuged at 3,000 rpm at 4°C for 15 min, 200 μl of the upper serum layer was transferred into a 1.5 ml centrifuge tube, and 2 μl of the protective agent was added. All steps were completed within 4 h, and blood samples were stored in a −20°C refrigerator for testing. Ultra-high-performance liquid chromatography-tandem mass spectrometry was used to quantitatively determine the concentrations of serum vitamin C. Firstly, internal standard working solution and calibration working solution were prepared. Subsequently, 60 μl serum and 60 μl internal standard working solution were poured into a 1.5 ml centrifuge tube, shaken for 5 min, and centrifuged at 12,000 rpm for 10 min. Thereafter, 70 μl supernatant was transferred into a 96-well sample. A 96-well sample plate covered with a silica gel plate was placed in the autosampler. The application software was started and the liquid chromatography and mass spectrometry conditions were set to establish the sample list. The vitamin C concentration of the serum sample was calculated and analyzed by the application software. The established vitamin C reference range was 6–25 μg/ml. Finally, the serum concentration of vitamin C in all participants was recorded.

### 2.3. Statistical analysis

Descriptive analysis was conducted. Continuous variables were tested for normality: normally distributed variables were analyzed using an independent sample *t*-test and variables that were not normally distributed were analyzed using the Mann–Whitney *U*-test. Categorical variables were analyzed using the Chi-square test. Subgroup analysis and Spearman’s rank correlation were used to analyze the relationship between vitamin C levels and related risk factors in the fracture group. All statistical analyses were performed using MedCalc® Statistical Software, version 20.106 (MedCalc Software Ltd, Ostend, Belgium). A two-tailed *p*-value < 0.05 indicated statistical significance.

## 3. Results

In total, 325 patients (200 boys, 125 girls) were included in the fracture group. The mean age was 5.1 years (95% CI, 4.83–5.33) (Table 1). All fractures were caused by mild or moderate trauma according to Clark’s classification. The most common type of fracture was upper limb fracture (86.2%), including supracondylar fractures of the humerus, lateral condyle fractures, and ulna and radius fractures. Femoral shaft and tibial fractures accounted for all lower limb fractures (13.8%). The control group included 318 patients (216 boys, 102 girls) with a mean age of 5.0 years (95% CI, 4.67–5.33) (Table 1). Diseases in the control group included tenosynovitis (21.7%), muscular torticollis (28.6%), skin lacerations (11.6%), and others (34.9%). There were no significant differences in age, sex, and BMI between the fracture and control groups (Table 1). Overall, the median serum vitamin C level was 4.48 µg/ml in the fracture group and 8.38 µg/ml in the control group (Mann–Whitney *U*-test, *p* < 0.0001) (Table 1).

**Table 1:** Comparison of patient characteristics between fracture and control groups.

	Fracture group (M, 95% CI)	Control group (M, 95% CI)	*p*-value*
Cases (*n*)	325	318	
Age (year)	5.1 (4.83–5.33)	5.0 (4.67–5.33)	0.0793
Sex (male/female)	200/125	216/102	0.0905^a^
BMI (kg/m^2^)	16.15 (15.70–16.42)	15.85 (15.60–16.00)	0.0522
Type of fracture (%)	Upper limb fractures (86.2)	N	
	Lower limb fractures (13.8)	N	
Non-fracture diseases (%)	N	Tenosynovitis (21.7)	
	N	Muscular torticollis (28.6)	
	N	Skin lacerations (11.6)	
	N	others (34.9)	
VC (µg/ml)	4.48 (3.93–4.97)	8.38 (7.83–9.24)	<0.0001

N, none; n, number; M, median; m, month; CI, confidence interval.

* Mann–Whitney *U*-test.

^a^ Chi-square test.

**Figure 1:**
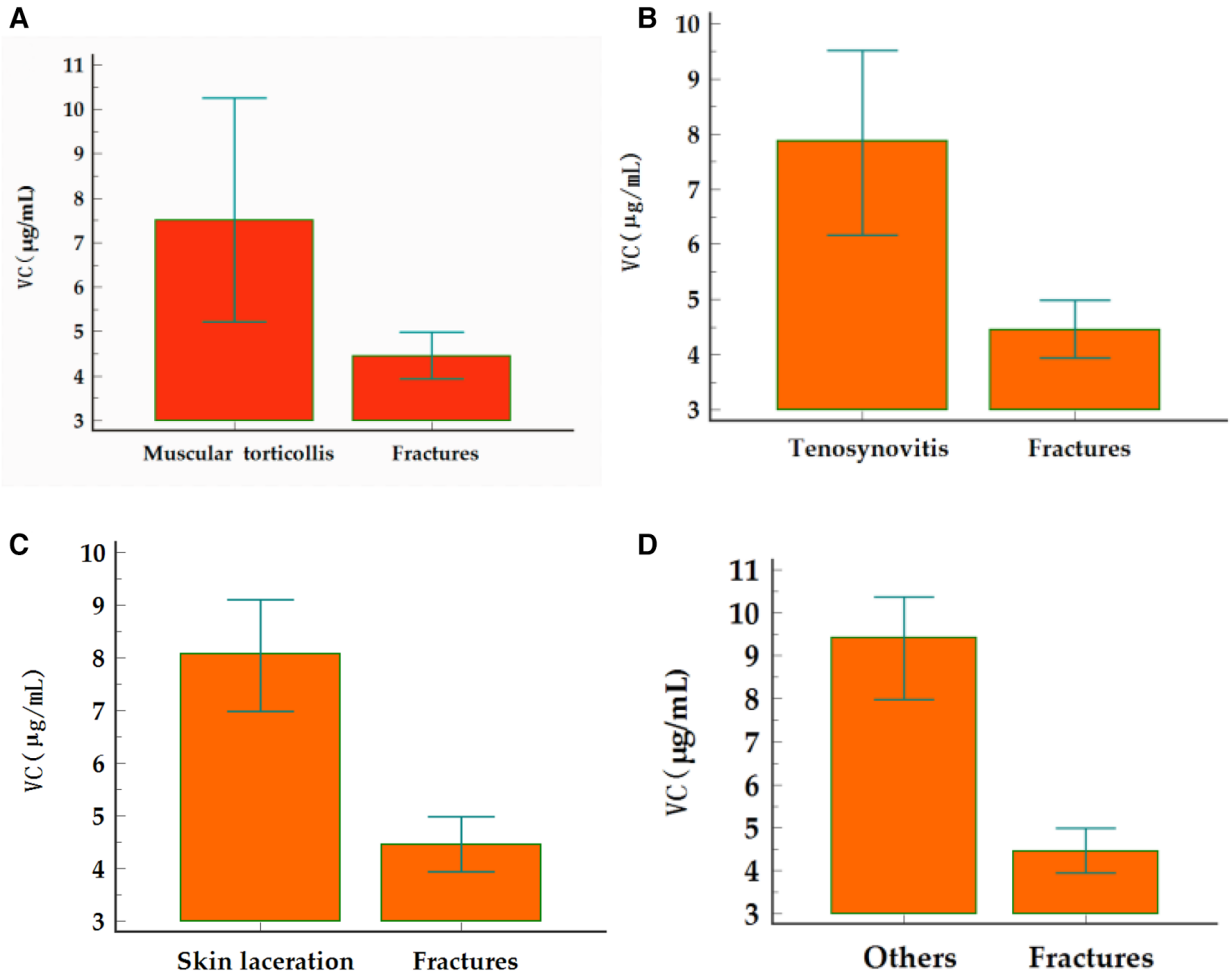
Comparison of serum vitamin C status between fracture group and non-fracture diseases in the control group. (**A–D**), muscular torticollis (Mann–Whitney *U*-test, *p* = 0.0026), tenosynovitis (Mann–Whitney *U*-test, *p* < 0.0001), skin laceration (Mann–Whitney *U*-test, *p* < 0.0001), and other diseases (Mann–Whitney *U*-test, *p* < 0.0001), respectively.

The fracture group was compared with the control group for various diseases including tenosynovitis, muscular torticollis, skin laceration, and other diseases (Figure 1). The serum vitamin C level was significantly lower in the fracture group than in the control group for all diseases.

We further analyzed the relationship between age, sex, BMI, duration of time after injury, injury site, and serum vitamin C levels in the fracture group (Table 2). The vitamin C level decreased after the age of 4 years, and was significantly different from that of the children under the age of 4 years (Mann–Whitney *U*-test, *p* < 0.05). There was a significant difference in the duration after injury between <6 and >6 h (Mann–Whitney *U*-test, *p* = 0.0224).

**Table 2:** Variations in vitamin C levels in the fracture group.

Variations	Vitamin C M (95% CI, µg/ml)	*p*-value*
Age (year)		
<4	5.51 (4.45–6.92)	0.0056
≥4	3.98 (3.56–4.81)	
Sex		
Male	4.64 (3.98–5.24)	0.8801
Female	4.30 (3.76–5.23)	
Duration after injury (h)		
≤6	4.08 (3.57–4.73)	0.0224
>6	5.52 (4.34–6.20)	
Fracture site		
Upper limb	4.32 (3.89–4.08)	0.4749
Lower limb	4.75 (3.79–614)	

* Mann–Whitney *U-*test; y, year; h, hour; M, median; CI, confidence interval.

**Figure 2:**
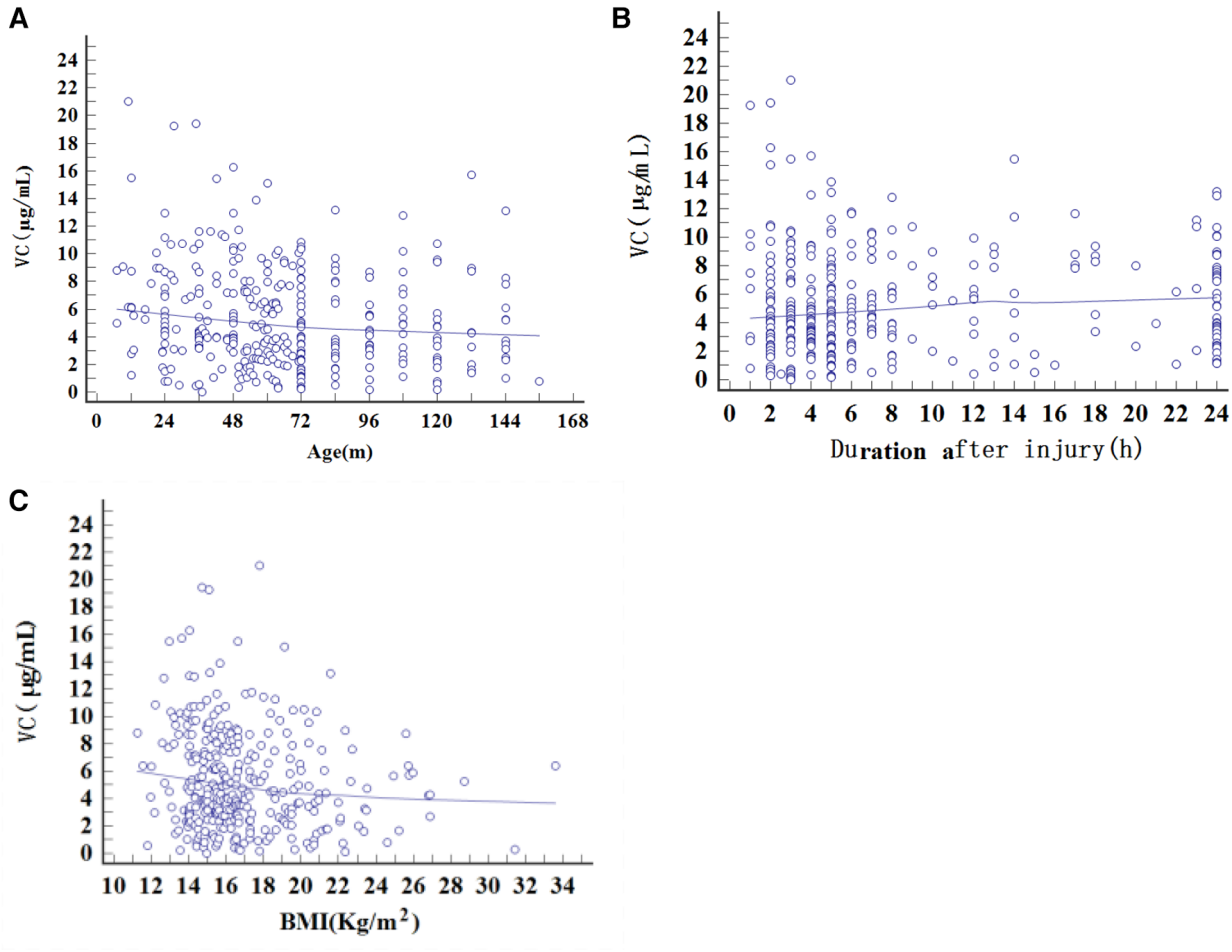
Spearman’s rank correlation coefficient analysis of factors regarding the change in vitamin C in the fracture group. (**A**) Age (*ρ* = −0.170, *p* = 0.0020); (**B**) Duration after injury (*ρ* = 0.0716, *p* = 0.1985); (**C**) BMI (*ρ* = −0.1446, *p* = 0.0091).

Spearman’s rank correlation coefficient was used to analyze the correlation between age, BMI, duration after injury, and vitamin C level in the fracture group (Figure 2). The results showed that age and vitamin C levels were negatively correlated (*ρ* = −0.170, *p* = 0.0020). BMI and vitamin C levels were also negatively correlate (*ρ* = −0.1446, *p* = 0.0091), while vitamin C levels were not significantly associated with the duration after injury.

## 4. Discussion

Previous studies have reported that Vitamin D is associated with pediatric limb fractures (16, 17). However, subsequent studies on serum vitamin C status after pediatric limb fractures have not attracted enough attention (20, 21). This study analyzed the serum vitamin C levels in children with limb fractures. We found that the vitamin C level in children with fractures was significantly lower than that in the control group. The serum vitamin C levels reported so far for healthy subjects are above 5 µg/ml (28 µmol/L) (4, 20), and our study showed the serum vitamin C level in children with limb fractures to be below this standard. This finding is similar to that in previous studies of adult fractures (13, 21, 22). For example, Falch measured the serum ascorbic acid concentration in 40 elderly patients with hip fractures caused by low-impact injuries and clarified that the serum ascorbic acid levels in older patients were relatively low (13).

Fracture may be a critical factor in the reduction of serum vitamin C levels. Blood vessels in the fracture area rupture to form a hematoma, resulting in the loss of serum vitamin C (23). However, owing to oxidative stress, a large amount of vitamin C is consumed at the fracture site. Oxidative stress is an imbalance between the production of oxidant and antioxidant species, with the disruption of redox signaling and/or molecular damage caused by overproduction of reactive oxygen species (ROS) (24). Animal experiments and clinical studies have shown that excessive activation of inflammatory cells produces a large number of ROS, leading to acute oxidative stress at fracture sites (8, 23). Vitamin C, as an antioxidant, participates in redox reactions to neutralize ROS and alleviate oxidative stress, which inevitably leads to the excessive consumption of vitamin C (23, 25). It is worth noting, however, that because insufficient intake of vitamin C can increase the risk of fracture (6), the possibility of vitamin C deficiency before fractures are sustained cannot be ruled out. Although the blood of the fracture group was collected within 24 h, it could not objectively reflect the serum vitamin C status of children before the fracture.

Recent research has shown that aging impairs the body’s ability to fight oxidative stress (25, 26). Older adults are more susceptible to oxidative stress than younger adults, and fractures can lead to higher levels of oxidative stress. Thus, fracture-related serum vitamin C levels may vary with age. In this study, the correlation analysis revealed that vitamin C levels negatively correlated with age; therefore, age may be considered an influencing factor for the reduction of serum vitamin C levels in pediatric fractures. We speculated that older children were more likely to experience more severe oxidative stress at the fracture site than were younger children, thus requiring more vitamin C consumption. Furthermore, the different dietary intake and nutritional status of infants and preschool-aged, school-aged, and adolescent children may result in different serum vitamin C levels before the fracture (27). Infants typically obtain a certain amount of vitamin C through the intake of dairy products. The diet of preschool-aged, school-aged, and adolescent children has diversified, and their vitamin C intake may be insufficient. Moreover, BMI is primarily affected by age (28); therefore, vitamin C levels are negatively correlated with BMI. Additionally, vitamin C levels may be influenced by the duration after injury (14). This study found that the level of vitamin C was significantly lower in the first 6 h and gradually increased over time in the later period. This indicates that the oxidative stress response intensely consumed vitamin C during the early fracture period. In the later stage, oxidative stress is weakened, thus reducing the consumption of vitamin C under the body’s compensatory function. Therefore, vitamin C deficiency after injury is likely to be temporary. A meta-analysis demonstrated that oral vitamin C supplementation did not improve first-year functional outcomes in orthopedic patients (5). Thus, the therapeutic effects of vitamin C on pediatric fractures need more research support.

Our study had some limitations. First, this study only examined fracture cases that occurred as a result of low-impact injuries that required surgical treatment but did not examine fractures treated conservatively in outpatient clinics. Second, despite rigorous screening, the control group was not representative of a healthy population. Finally, although the serum vitamin C in the fracture group was deficient according to the reference range (6–25 µg/ml) set by the kit, this requires sufficient supporting clinical data.

## 5. Conclusions

This study revealed that serum vitamin C levels were significantly lower in children with limb fractures than they were in children without fractures, and were generally below the normal range. Oxidative stress and acute depletion after fracture might be the main factors. Vitamin C insufficiency was more likely to be present in children with limb fractures who were older than 4 years and within 6 h after injury. However, vitamin C insufficiency after fracture may be temporary, and further research is needed to address this.

## Data Availability

The raw data supporting the conclusions of this article will be made available by the authors, without undue reservation.
